# Metalized polyamide heterostructure as a moisture-responsive actuator for multimodal adaptive personal heat management

**DOI:** 10.1126/sciadv.abj7906

**Published:** 2021-12-15

**Authors:** Xiuqiang Li, Boran Ma, Jingyuan Dai, Chenxi Sui, Divya Pande, David R. Smith, L. Catherine Brinson, Po-Chun Hsu

**Affiliations:** Thomas Lord Department of Mechanical Engineering and Material Science, Duke University, Durham, NC 27708, USA.

## Abstract

Personal thermal management textile/wearable is an effective strategy to expand the indoor temperature setpoint range to reduce a building’s energy consumption. Usually, textiles/wearables that were engineered for controlling conduction, convection, radiation, or sweat evaporation have been developed separately. Here, we demonstrate a multimodal adaptive wearable with moisture-responsive flaps composed of a nylon/metal heterostructure, which can simultaneously regulate convection, sweat evaporation, and mid-infrared emission to accomplish large and rapid heat transfer tuning in response to human perspiration vapor. We show that the metal layer not only plays a crucial role in low-emissivity radiative heating but also enhances the bimorph actuation performance. The multimodal adaptive mechanism expands the thermal comfort zone by 30.7 and 20.7% more than traditional static textiles and single-modal adaptive wearables without any electricity and energy input, making it a promising design paradigm for personal heat management.

## Teaser

A wearable device that responds to sweat vapor can switch between radiative heating and evaporative cooling.

## INTRODUCTION

Building energy consumption currently contributes more than 30% of global energy consumption and ~20% of global greenhouse gas emissions (*1*, *2*). Among these, half of the energy is used for building heating and cooling management. Moreover, owing to the rapid population growth and climate change, it is predicted that the percentage for heat management will continue to grow toward ~80% by the end of 2050 (*1*, *2*). The fundamental reason for the enormous energy consumption lies in our demand for thermal homeostasis that is indispensable for health and productivity. Creating innovative solutions to overcome this energy-health dilemma has become a critical research topic for scientists and engineers in recent years. Personal thermal management based on smart textile/wearable is a promising and effective strategy to reduce heating, ventilation, and air conditioning (HVAC) energy consumption by focusing on the local environment around the human body instead of the entire building interior space (*3*–*5*). For example, Ghahramani *et al.* (*6*) reported that the baseline setpoint of 22.5°C can be expanded ±1°, ±2°, and ±3°C after using personal thermal management technologies, and correspondingly, HVAC energy savings could reach 7.5, 12.7, and 16.4%, respectively.

The heat transfer between the human body and environment through clothing mainly includes four mechanisms: conduction and convection, radiation, and sweat evaporation. On the basis of different forms of heat transfer, various passive smart textiles/wearables have been developed to regulate heat conduction and heat convection (*7*–*9*), heat radiation (*10*–*15*), or sweat evaporation (*16*–*20*) to achieve personal thermal management. To enhance the functionality, sweat-responsive thermal regulation strategies have been demonstrated, which can automatically adjust the heat transfer coefficients in response to sweat vapor (*21*–*25*). These adaptive textiles/wearables have the tunability advantages as active thermal textiles/wearables but with minimal or even zero energy consumption, making them a promising new approach for personal thermal management (*10*). Currently, sweat-responsive thermoregulation can be divided into two categories. One is based on the bicomponent fibers that can convert the yarns between tight and loose forms. The other is based on the opening and closing of flaps. Compared with fiber actuators, the flap bimorph actuation has a more extensive tuning range due to the larger effective area. However, the reported moisture-responsive materials for flap configuration are much more costly than traditional textiles, and the tuning mechanisms are solely based on convection, both of which hinders further thermoregulation performance improvement. In particular, the potential of mid-infrared (mid-IR) management accounting for approximately 50% of the heat dissipation of our human body through radiation in typical indoor conditions (*12*) has been overlooked. A much more extensive tuning range and more functionalities could be created if multiple heat transfer mechanisms can be incorporated to work synergistically as a “multimodal wearable.” A comparison of current sweat-responsive thermoregulation technologies can be found in table S1.

**Fig. 1.**
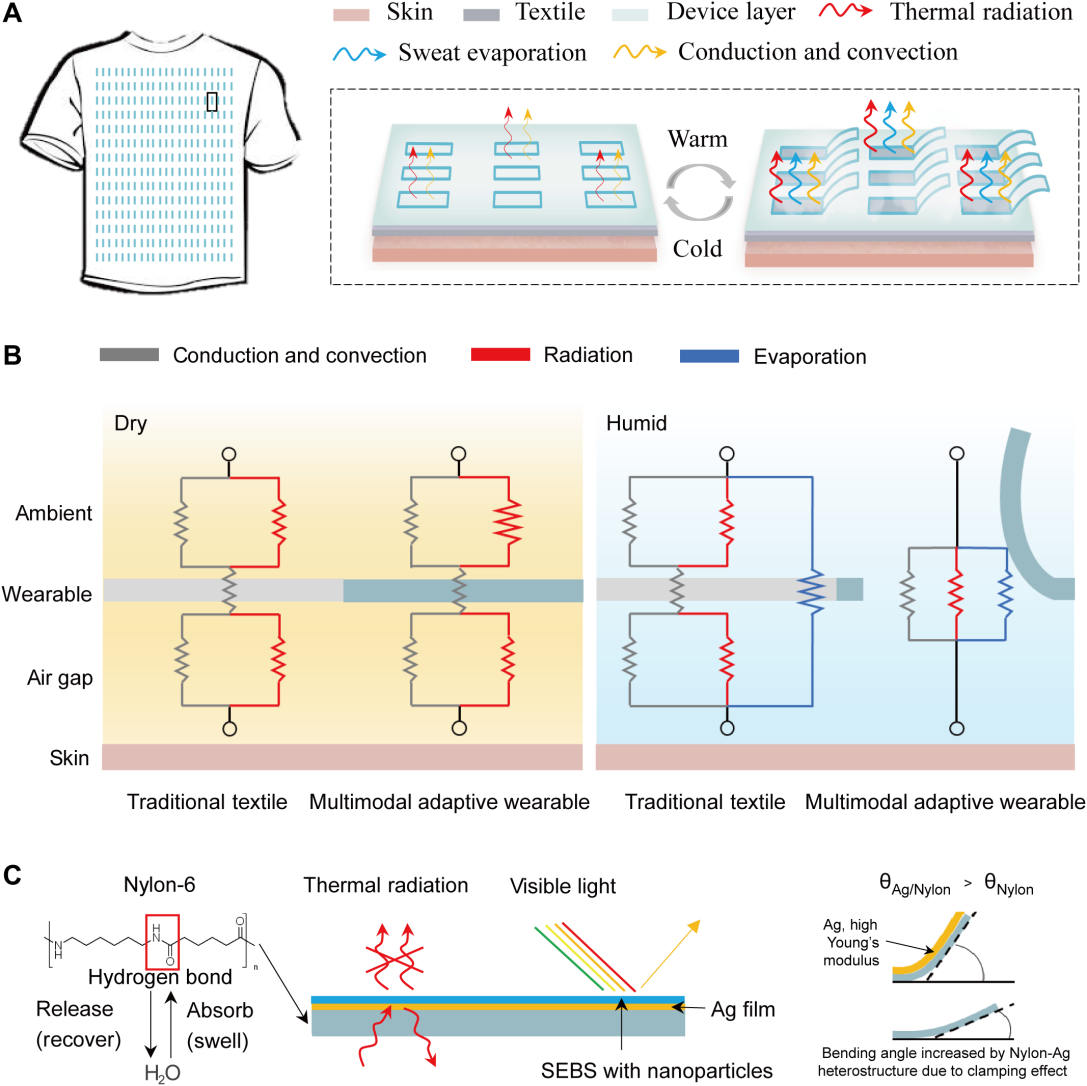
Concept and mechanism of nylon-Ag heterostructure moisture-responsive wearable. (**A**) Working principle of nylon/Ag heterostructure–based multimodal wearable. (**B**) Analysis of thermal resistance of traditional textile and our designed moisture-responsive wearable in dry (left) and humid (right) environments. (**C**) Composition of the multimodal adaptive wearable: configuration of the nylon-Ag–SEBS heterostructure and the functional diagram of each layer. Bending of nylon-Ag actuator is augmented due to the clamping effect: Smaller strain at the interface (compared to the unrestrained bottom surface of nylon) caused by depositing Ag layer leads to increasing bending angle.

In this work, we demonstrate a multimodal smart wearable with moisture-responsive flaps composed of metalized nylon heterostructure, which can simultaneously regulate convection, mid-IR emission, and sweat evaporation by rational multifunctional designs of materials with proper mechanical, optical, and wearable properties (Fig. 1A). A polystyrene-block-poly(ethylene-ran-butylene)-block-polystyrene (SEBS) nanocomposite is used as the top passivation layer. As shown in Fig. 1B (left), compared with traditional textile, our design with dry state features a low-emissivity layer that can suppress the radiation heat loss to achieve effective heating. In the humid state, the flaps open automatically to promote convection, radiation, and sweat evaporation (see Fig. 1B, right) for cooling. The result showed a significant expansion of thermoregulation capability compared to traditional textiles and nonmetallized nylon by 30.7 and 20.7%, respectively. Last, we demonstrate that SEBS nanocomposite can provide an added feature of different colors without compromising the performance of thermal management.

## RESULTS AND DISCUSSION

To achieve the multimodal thermal management, three layers (nylon, silver, and SEBS nanocomposite; see Fig. 1C) with an array of flaps (Fig. 1A) were carefully designed.

### Nylon-6 layer

As one of the most widely used polyamides, nylon-6 can reversibly absorb and release water to achieve bimorph actuation by hygroscopic expansion. Specifically, the nylon flaps will bend toward the lower humidity when there is a humidity difference between the two sides and recover to their original state when the humidity difference vanishes. This humidity sensitivity is attributed to the amide (─CONH) group in the nylon chains, which can form hydrogen bonds with water molecules (see Fig. 1B, left) (*26*, *27*). The opening and closing of these flaps can tune the convective and evaporative heat exchange between our human body and the ambient air (Fig. 1A). Compared with other reported moisture-responsive materials such as Nafion film (*21*, *24*), graphene oxide (GO) film (*28*), reduced GO film (*29*), and MXene film (*30*), nylon is one of the most common textile materials due to its low cost, high stability, and excellent mechanical properties, and it also has the advantage of mid-IR transparency (discussed below).

### Ag layer

To incorporate radiative heat tuning to the nylon moisture-responsive actuator, a layer of Ag was deposited on the top surface to achieve low mid-IR emissivity. This layer effectively suppresses radiation loss when the flaps are closed in the dry state. Conversely, when the flaps are open under humid condition, radiative heat transfer increases because human skin is close to a perfect black body. It is found that the nylon film’s actuation performance can be notable enhanced by properly choosing the thickness of Ag film due to the clamping effect (see Fig. 1C, right).

### SEBS nanocomposite

To increase the options of the wearable’s visible appearance for aesthetic purposes (*31*), a top color layer was designed. This color layer needs to be mid-IR transparent to maintain the design’s low emissivity. After careful analysis and screening, we chose the combination of SEBS polymer with Si, Fe_2_O_3_, and CuO nanoparticles, which can serve as effective mid-IR transparent coloration layers deposited by spray coating. We will further discuss these designs in the following paragraphs. The samples of the nylon-Ag actuators are labeled with Ag layer thickness indicated, for example, Nylon-Ag50 refers to the sample with 50-nm-thick Ag deposited on nylon.

**Fig. 2.**
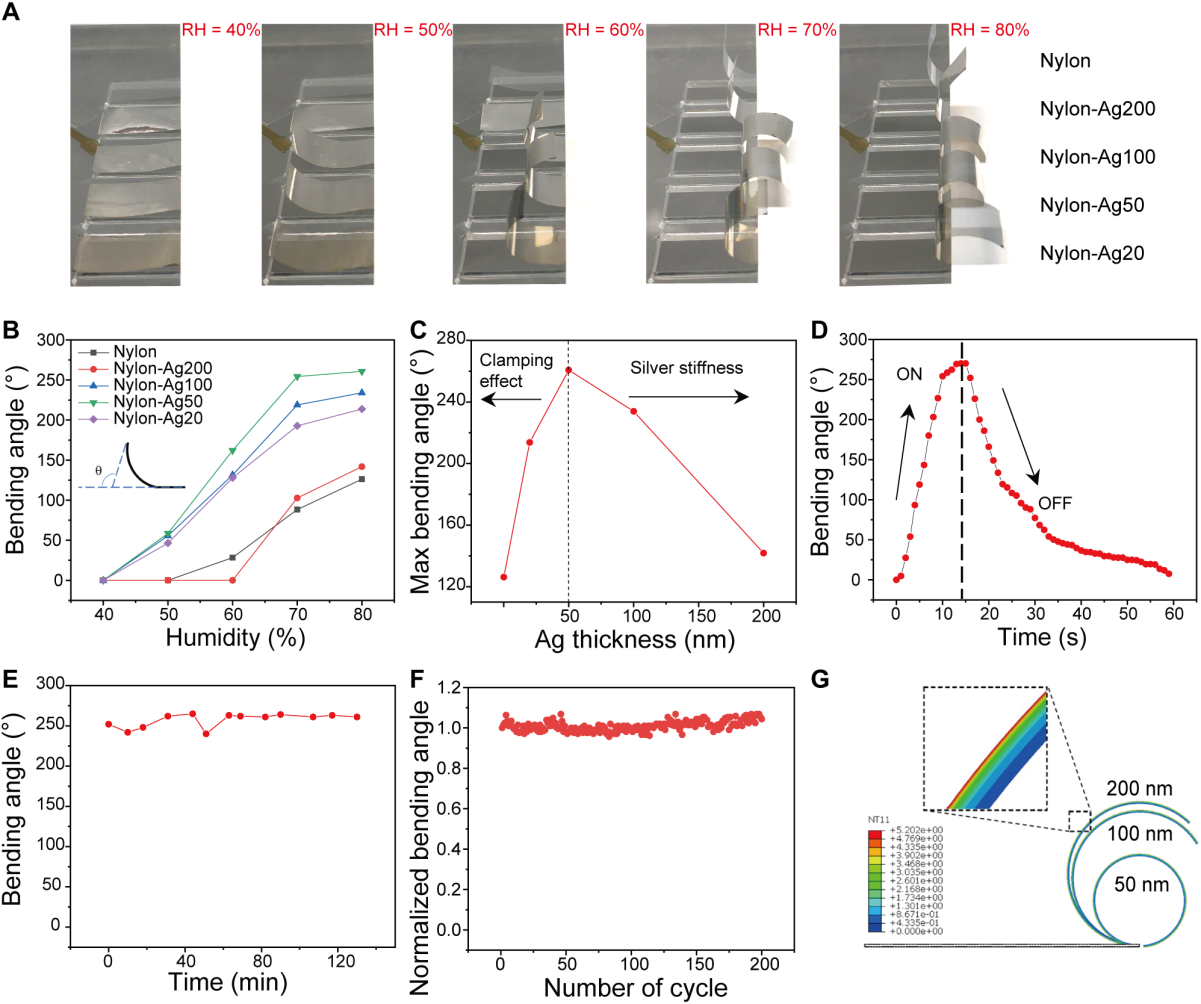
Performance of nylon-Ag heterostructure–based moisture-responsive actuator. (**A**) Photos of the bending process of nylon-Ag actuators (length, 2 cm; width, 1 cm) over different humidities. (**B**) Bending angle of nylon-Ag actuator with different thicknesses of Ag as a function of humidity (inset, definition of bending angle.). (**C**) Maximum bending angle versus the thickness of Ag. (**D**) Dynamic behavior of bending and recovery in response to moisture. The sample is nylon-Ag50. (**E**) Bending angle versus time at RH = 80%. (**F**) Cycle performance of nylon-Ag50 actuator. The closing angle of each cycle is less than 5°. (**G**) FEA simulations of nylon-Ag bilayer film with various Ag layer thicknesses; color legend shows the temperature field (which corresponds to the water content gradient in the simulation) across the film. Photo credit: (A) Xiuqiang Li, Duke University.

To investigate the moisture-responsive properties of nylon-Ag actuator, as shown in Fig. 2A, a quantitative characterization of nylon’s bending process with different thicknesses of Ag over humidity was carried out. As shown in Fig. 2B, the bending angle of Nylon-Ag50 can reach 260° when moisture is introduced and increases the local humidity below the nylon film from 40 to 80%. Intriguingly, as shown in Fig. 2C, the maximum bending angle exhibits a nonmonotonic trend, which increases initially as the Ag thickness increases and reaches the maximum at 50 nm before decreasing. Fundamental analysis is detailed in the next paragraph. In addition to the bending angle, the device’s response time and stability are also critical figures of merit. As shown in Fig. 2D, Nylon-Ag50 can be fully opened in ~14 s when the relative humidity (RH) changes from 40 to 80% and return to the initial state at ~45 s when the RH gradient disappears (the corresponding optical images can be seen in fig. S1). If the humidity is maintained at 80%, the actuator’s bending angle is unchanged for 2 hours (Fig. 2E). Apart from having good bending properties and response time, Nylon-Ag50 also exhibits excellent stability showing an unchanged bending angle after 200 cycles (Fig. 2F). Moreover, two-dimensional (2D) wide-angle x-ray scattering patterns of nylon films also demonstrate no apparent preferred orientation of the nylon film, so it can be treated as an isotropic semicrystalline film for analysis (fig. S2).

To understand the effect of the deposited Ag nanolayer on the hygroscopic behavior of the nylon film, simplified analytical mechanical modeling was carried out. Defining λ as the expansion ratio of the nylon film upon moisture absorption, *t*_1_ and *t*_2_ as thickness of Ag and nylon layers, respectively, mechanical equilibrium requires that the net forces and moments of the bilayer structure are zero. This constraint yields the absolute value of the radius of curvature at equilibrium (details in note S1)

$$|R|=\frac{A(1-\lambda )t_{1}^{4}+2(2+\lambda )t_{1}^{3}t_{2}+3(2+\lambda )t_{1}^{2}t_{2}^{2}+4t_{1}t_{2}^{3}+\frac{1}{A}t_{2}^{4}}{6\lambda t_{1}t_{2}(t_{1}+t_{2})}$$ (1)

where *A* = *E*_1_/*E*_2_, *E*_1_ and *E*_2_ are modulus of Ag and nylon layers, respectively. A smaller value of ∣*R*∣ corresponds to a larger bending angle. The equation above provides a numerical explanation of the impact of a thin layer of high modulus material on the bending behavior of the nylon film. First, note that the magnitude of the modulus ratio, *A*, is ~138, because Ag has a Young’s modulus of ~83 GPa (*32*) and the nylon film’s Young’s modulus is ~0.6 GPa, as measured in experiments with water absorbed. Therefore, the contribution from the *t*_2_ term is decreased, while the contribution from the *t*_1_ term is increased, both by a factor of ~138; because *t*_2_ ≫ *t*_1_, the overall effect is a reduction in the radius of curvature, i.e., an augmented bending of the nylon film. From a physical sense, because of the competition of the swelling of the nylon versus the restraint of the Ag layer, at the interface of Ag and nylon, the Ag coating undergoes expansion, whereas nylon film experiences contraction. Therefore, a high Young’s modulus of the coating layer gives rise to a smaller amount of strain at the interface (compared to the unrestrained swelling strain at the lower surface of the nylon), which results in an increase in bending angle of the composite film.

The analytical result also shows a nonmonotonic trend in the thickness-dependent hygroscopic behavior of the nylon-Ag film (see note S1), which agrees with the trend observed in the experiments (Fig. 2C). The substantial thickness contrast and stiffness differential between the two layers results in a resistance to bending at both limits of the Ag layer thickness, leading to the nonmonotonic trend mentioned above and therefore an optimum thickness of Ag layer for the nylon-Ag actuator. However, the optimum thickness of the Ag layer obtained by the analytical model still has discrepancy from the experimental result; even when considering a gradient water concentration within the nylon film, this could be partially due to the variation in the Young’s modulus of the Ag layer from that reported in (*32*), resulting from a different synthesis method. Therefore, finite element analysis (FEA) was performed to further investigate this discrepancy. FEA simulations of a 2D bilayer structure have validated our simplified analytical model for the cases where the two layers have similar Young’s moduli (e.g., 83 and 20 GPa), whereas for cases where the two layers have a larger Young’s modulus mismatch, the analytical model is not fully able to capture the Ag layer thickness dependence of the bending behavior of the bilayer structure. The lack of accuracy of the analytical model may arise from omission of the shear forces between the two layers.

A series of 2D FEA simulations of bilayer structures that characterize the mechanical properties of both nylon and Ag layers were carried out using ABAQUS. The thermal expansion analysis included in ABAQUS was used as an analog for the hygroscopic behavior of the nylon film. One end of the bilayer film is fixed, as in the experimental setup (Fig. 2A), and a temperature gradient is used to mimic the humidity gradient (see note S1). As shown in Fig. 2G, one can see that as the thickness of Ag layer decreases, the bilayer film bends more with Nylon-Ag50 leading to the largest bending angle, aligning with the optimum thickness observed in experiments. According to FEA simulations, the bilayer film starts showing reduction in bending as the Ag layer thickness decreases from 50 to 40 nm (see table S2), replicating the nonmonotonic trend of the analytical model and experimental data.

**Fig. 3.**
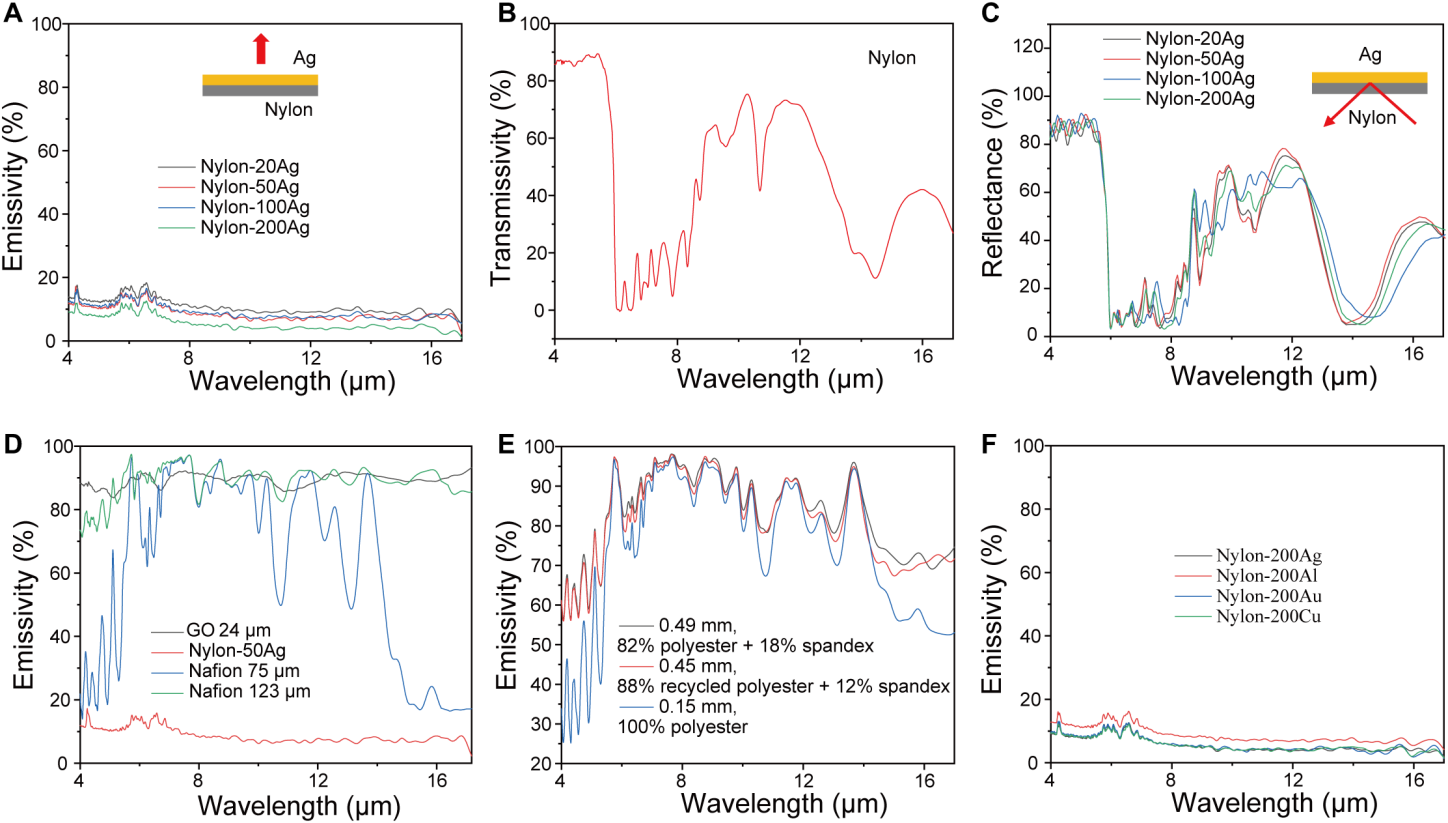
Mid-IR performances of nylon-Ag heterostructure–based moisture-responsive actuators. (**A**) Mid-IR emissivity of nylon-Ag actuators (from the Ag side). (**B**) Mid-IR transmissivity of nylon film. (**C**) Mid-IR reflectance of nylon-Ag actuators (from the nylon side). (**D**) Mid-IR emissivity of other exemplar moisture-responsive actuator materials. (**E**) Mid-IR emissivity of traditional textiles. (**F**) Mid-IR emissivity of nylon with different metals.

As mentioned above, the metal layer not only improves nylon’s bending performance but also suppresses the IR emission of the human body. Figure 3A shows the mid-IR emission of nylon-Ag actuator. It can be seen that even with only a 20-nm-thick Ag layer, its emissivity can be reduced to only 0.1, which is around eight to nine times smaller than traditional textiles and other stimuli-responsive materials, resulting in superior heating effect in the dry state. In addition, pristine nylon has ~44% transparency in mid-IR (Fig. 3B), so the top Ag layer can also increase the mid-IR reflectivity from below, which directly reflects and traps human body’s thermal radiation to achieve even stronger thermal insulation effect (Fig. 3C) (*14*). Because the Ag layer is opaque to mid-IR, one can also interpret the heating effect by the low emissivity probing from the nylon side. It is worth noting that this low mid-IR emissivity has not been observed in other material systems, e.g., GO and Nafion (Fig. 3D), and traditional textiles (Fig. 3E). Also, it can be shown that other metals behave very similarly with Ag in mid-IR. For example, the mid-IR emissivity of Au, Al, and Cu can all reach <10% (Fig. 3F), indicating the universality, cost-effectiveness, and scalability of the metal/nylon approach.

**Fig. 4.**
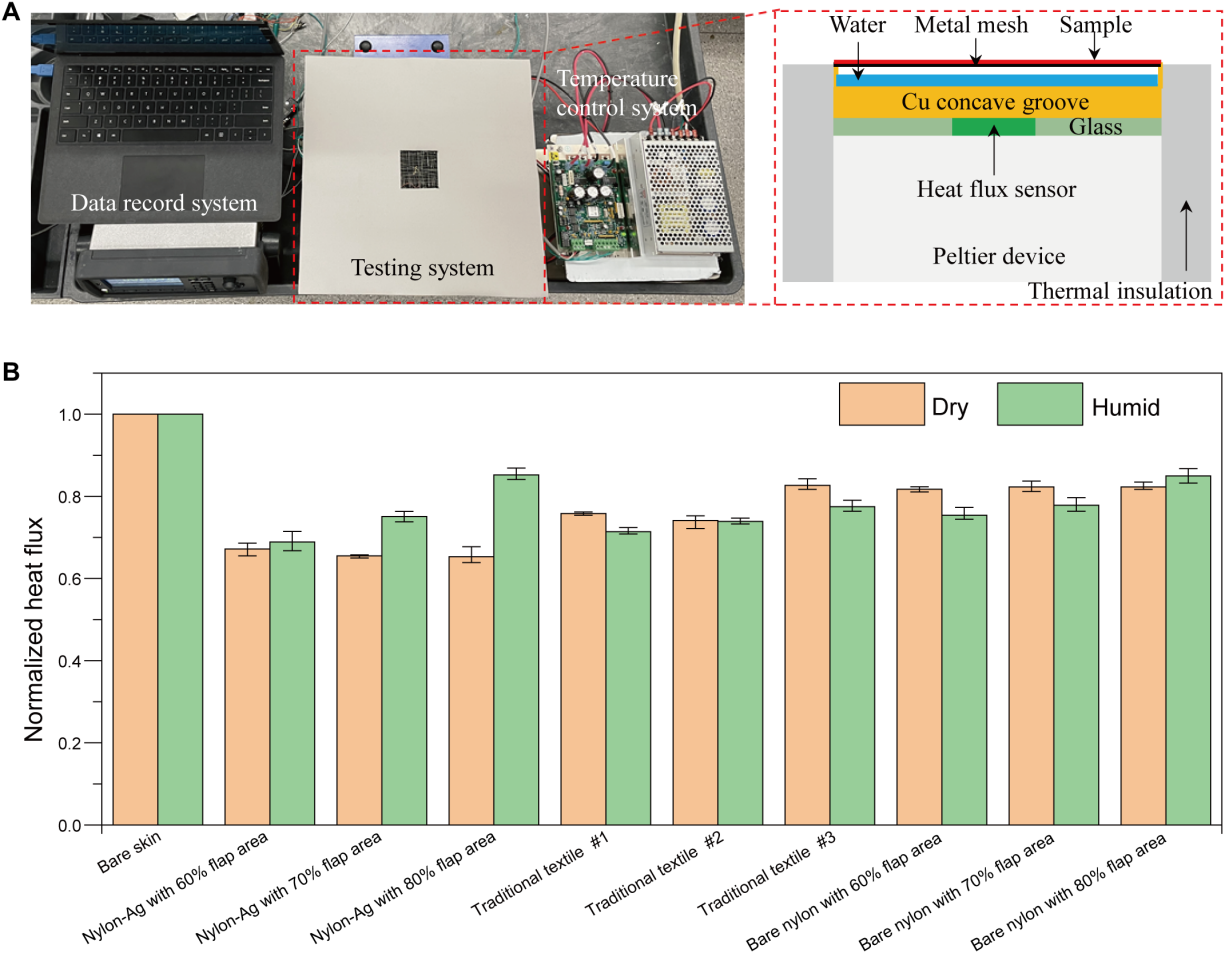
Thermal performances of nylon-Ag heterostructure–based moisture-responsive actuators. (**A**) Photo of the overall system and schematic of testing setup. (**B**) Normalized heat flux of different types of textiles/wearables at dry and humid state. The thickness of nylon-Ag and bare nylon wearables is 17 μm (Ag thickness is negligible). Traditional textile #1, 82% polyester + 18% spandex (490 μm thick); traditional textile #2, 88% polyester + 12% spandex (450 μm thick); traditional textile #3, 100% polyester (150 μm thick). The error bars in (B) represent the SD of three independent measurements. Under humid test conditions, the RH under flaps is >95%. Photo credit: (A) Xiuqiang Li, Duke University.

The proper design of mid-IR and actuation properties leads us to conduct heat transfer measurements to quantify the multimodal adaptive heat management performance. As shown in Fig. 4A, the testing system features a Peltier temperature control feedback system including temperature control system and testing system (see Fig. 4A, left) and a data recording system. In this system, a proportional integral derivative (PID) control program is used to fix the carbon-coated copper concave groove’s temperature with/without water to simulate the human skin at humid/dry state (under humid conditions, RH > 95%). At steady state, the heat flux supplied by the Peltier device equals the heating or the cooling generated by simulated skin with cloth, measured by the heat flux sensor between the Peltier device and the copper concave groove (see Fig. 4B, right; more details can be found in Materials and Methods). For fair comparison and data interpretation, we normalized the heat flux based on the bare skin, i.e., no wearable sample. Ideally, to help the user adapt to large ambient temperature fluctuation, the adaptive wearable should suppress the normalized heat flux at dry state (flaps closed, fig. S3A) and increase the normalized heat flux at humid state (flaps open, fig. S3B). As shown in Fig. 4B, at dry state (indicated as yellow), the normalized unitless heat flux of traditional textiles #1, #2, and #3 is 0.76, 0.74, and 0.83, respectively, and the three bare nylon samples with different flap areas all show 0.82 of normalized heat flux. In contrast, nylon-Ag wearables effectively suppressed the heat loss because of the radiative heating design, which achieves 0.65 of normalized heat flux. Note that this notable heating performance is achieved by the nylon-Ag wearable that is only 17 μm thick, which is approximately 20 times thinner than traditional textiles. This surface-sensitive characteristic is also one of the unique advantages of radiative heat management. Also, in Fig. 4B, the blue bars show the heat fluxes normalized to humid skin. Traditional textiles #1, #2, and #3 are 0.71, 0.74, and 0.78, respectively. For both bare nylon and nylon-Ag wearables, the moisture-responsive actuation allows convection and evaporation, so the heat flux of cooling performance increases as the flap area increases. In particular, the normalized heat flux of nylon-Ag wearable with 80% flap area can reach 0.85, which is the largest among all samples. Compared with traditional textiles, on average, the nylon-Ag wearable is warmer by 16.3% in the dry state and cooler by 14.4% when humid. In other words, the nylon-Ag wearable can expand the thermal comfort zone by 30.7%. As for the bare nylon, although it is capable of moisture-responsive thermoregulation, it is still outperformed by the nylon-Ag wearable by 20.7%. The difference mainly lies in the heating performance at dry state, which indicates the importance of incorporating radiation into thermal engineering. This large tunability demonstrates the efficacy of using multimodal design for adaptive heat management to achieve extended-range thermal comfort stabilization.

**Fig. 5.**
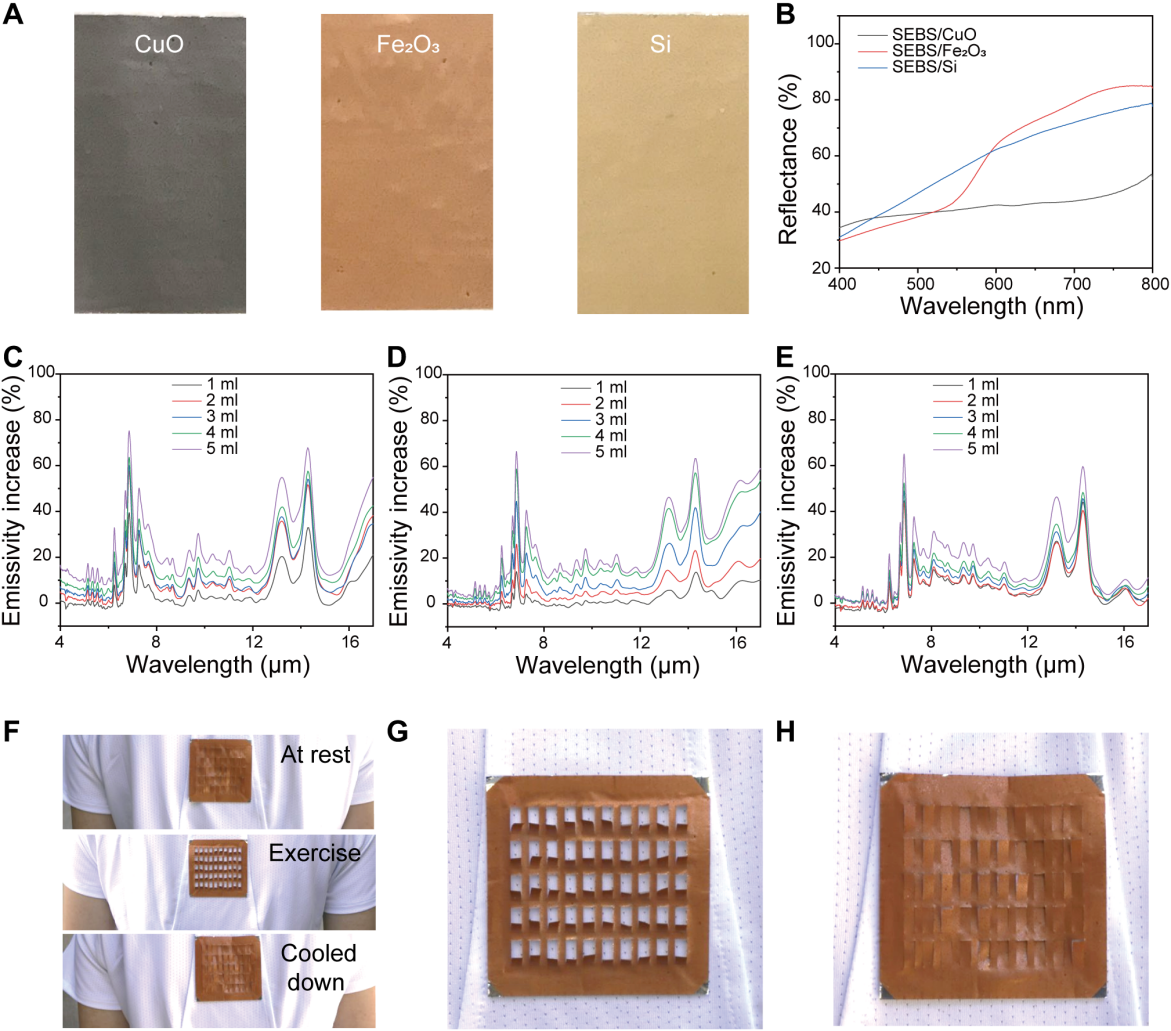
Design of colored wearables and outdoor experiment. (**A**) Photos of nylon-Ag wearable with SEBS nanocomposites of CuO/SEBS, Fe_2_O_3_/SEBS, and Si/SEBS. (**B**) Visible reflectance spectra of nylon-Ag wearable (6 cm by 6 cm) with CuO/SEBS (5 ml), Fe_2_O_3_ nanoparticles/SEBS (5 ml), and Si nanoparticles/SEBS (5 ml). (**C** to **E**) Emissivity increases of nylon-Ag wearable with different amounts of CuO/SEBS, Fe_2_O_3_/SEBS, and Si/SEBS, respectively. (**F**) Photos of wearable (6 cm by 6 cm) before, during, and after exercise. The moisture-responsive actuator switch between flat and curved well with the human sweat vapor in real situations. (**G** and **H**) Enlarged views of the wearable at the curved (during exercise) and flat (cooled down) states. Photo credits: (A) Xiuqiang Li, Duke University; (F to H) Chenxi Sui, Duke University.

In addition to thermal management, the color design for wearables is also an important factor for practical use. However, it is nontrivial to simultaneously achieve low emissivity and visual color because traditional organic dyes have strong absorption in mid-IR, e.g., C─O stretching (7.7 to 10 μm), C─N stretching (8.2 to 9.8 μm), aromatic C─H bending (7.8 to 14.5 μm), and S═O stretching (9.4 to 9.8 μm) (*33*). After careful analysis and screening, we found that the combination of SEBS with CuO, Fe_2_O_3_, and Si nanoparticles can achieve different colors while maintaining the low emissivity because of the nanocomposites’ high transmissivity in mid-IR. As shown in Fig. 5A, black, brown, and light yellow can be achieved by doping CuO, Fe_2_O_3_, and Si nanoparticles, respectively (the corresponding visible reflection can be found in Fig. 4B and fig. S4). The color depth is adjusted by varying the concentration of SEBS nanocomposites (fig. S5). As shown in Fig. 5 (C to E), the color layer design only slightly increases the mid-IR emission of the wearable. Similarly, the device’s bending performance was also well maintained (fig. S6). Last, the device was attached to the backside of a commercially available T-shirt (back) for outdoor experiments on a volunteered human subject. As shown in Fig. 5F (top), the flaps of the device are in the closed state at the beginning. When the subject starts to exercise (~5 min), the device’s flaps are fully opened (Fig. 5, F, middle, and G). As expected, when the subject stopped exercising and the sweat vapor fully disappeared, the device’s flaps returned to the initial closed state (Fig. 5, F, bottom, and H). Note that this demonstration is to show the attributes of its moisture response under real conditions. Other ways of usage, such as direct contact with the skin (*22*) or attaching to a very thin textile, are also possible.

In summary, we demonstrate a new metalized nylon-based multimodal smart wearable with sweat vapor–actuated low-emissivity flaps, which can synergistically regulate convection, mid-IR emission, and sweat evaporation from the human body to the ambient air. With rational designs of optical and mechanical properties, we demonstrated that the nylon-Ag wearable could significantly expand the adaptability by 30.7% more than traditional static textiles. In the future, we anticipate that large-scale manufacturing can be achieved by the metallization tools widely used in packaging industry, such as antistatic bags and oxygen-blocking films. We also envision that with further development of advanced materials, printing process, patterning techniques, and dynamic designs, the multimodal adaptive wearables will bring immense opportunities for energy efficiency and wearable technology.

## MATERIALS AND METHODS

### Preparation of nylon-Ag film and nylon-Ag/SEBS with nanoparticles

The silver film with different thicknesses was deposited onto the nylon film (17 μm thick, Goodfellow Company) using an evaporator (Kurt Lesker PVD 75). For the preparation of nylon-Ag/SEBS with nanoparticle film, 5 weight % (wt %) SEBS (molecular weight = 89,000; Sigma-Aldrich) solution was prepared by dissolving SEBS in hexane (Sigma-Aldrich) at 60°C. Then, 0.1 g of silicon nanoparticles (99%, 100 nm, SkySpring Nanomaterials Inc.), 0.5 g of iron oxide nanoparticles (99%, 20 to 40 nm, SkySpring Nanomaterials Inc.), and 0.5 g of copper oxide nanoparticles (99%, 40 nm, SkySpring Nanomaterials Inc.) were added to 50 g of 5 wt % SEBS solution. After sonication for 20 min, 5, 4, 3, 2, and 1 ml of the colored solution were sprayed by using an airbrush gun (PB-KTG, Fy-Light) to the Ag film side of the nylon-Ag film (6 cm by 6 cm), which was heated to 60°C on a hot plate. Last, the sample was heated for another 10 min to ensure evaporation of hexane. A rectangular array of flaps on the nylon-Ag film or nylon-Ag/SEBS with nanoparticles was cut using a razor blade.

### Characterizations

The reflectance of the nylon-Ag film and nylon-Ag/SEBS with nanoparticles was measured with an ultraviolet (UV)–visible near-IR spectrometer with a calibrated BaSO_4_ integrating sphere (300 to 1600 nm, Agilent Technologies, Cary 6000i) and the Fourier transform IR spectrometer with a diffuse gold integrating sphere (4 to 17 μm, Thermo Fisher Scientific, iS50). Sample surfaces were analyzed with a 3D noncontact surface profiler, Zygo New View 5000 (Zygo, Middlefield, CT, USA). The nylon films were characterized by x-ray diffraction (XRD), using the Panalytical X’Pert PRO MRD HR XRD System, with CuKα radiation (λ = 1.5418 Å), 40 kV, 40 mA, and scanning 2θ from 10° to 30° at a scanning rate of 0.05°/s. Wide-angle x-ray scattering data were measured using a SAXS Lab Ganesha instrument (SAXS LAB ApS, Skovlunde, Denmark), which is a point-collimated pinhole system, and a 2D Dectris Pilatus 300k 20-Hz detector equipped with Xenocs Genix ULD SL X-ray Source operating at 50 kV. During the measurement of scattering intensity, source-to-detector distances were set to be able to cover the wave vector range 0.07 < *q* < 2.8 Å^−1^. The 2D scattered pattern was monitored using the detector and then radially averaged using SAXS Gui software.

### Sample bending curvature measurement

A humidity-controlled chamber was built for the sample bending curvature measurement (as shown in fig. S7). Low-humidity and high-humidity air supplies were connected to the chamber and controlled by two valves. The chamber’s top surface was not sealed and had 1 cm by 2 cm holes, allowing samples to be exposed to different humidity environments. A digital humidity sensor (SEK-SHT31-Sensors, Sensirion Co. Ltd.) was placed right under the sample to measure humidity and temperature simultaneously. The resolution is 0.01% RH and 0.01°C, respectively. For bending curvature measurement of nylon with different Ag film thicknesses, humidity in the chamber was adjusted from 40 to 80%. Photos of the samples at different humidity levels were taken with a camera positioned at the same level as the samples.

### Heating power and cooling power measurement

Figure 4A shows the schematic of equipment, which can quantitatively test the heating power and cooling power. From the top surface, it involves metal mesh, copper concave groove (width, 5 cm; length, 5 cm; thickness, 4 mm; groove depth, 3 mm; the inner side of groove was coated with a layer of carbon to simulate the emissivity of human skin; see fig. S8) with/without 1 to 2 mm height of the water, heat flux sensor of 1 mm thick (Electro Optical Components Inc., A-04457) surrounded by glass with the same thickness, and a PID-controlled Peltier device (TE Technology Inc., TC-36-25). Thermal grease (Dow Corning, 340) was applied to ensure good thermal contact among different parts. The testing equipment’s sidewall is wrapped with polyurethane foam of about 5 cm thick to avoid heat losses. During the testing process, the copper plate’s temperature is kept at 33°C by using the PID program to simulate the human skin temperature, and the ambient temperature is 23°C. At steady-state, the supplied heating power compensates for the copper plate’s heat dissipation, and the heat flux sensor measures the corresponding cooling power or heating power in watts per square meter.

## Supplementary Materials

### This PDF file includes:

Supplementary Text Figs. S1 to S13 Tables S1 and S2 References
