# Supplementary material for: Metalized polyamide heterostructure as a moisture-responsive actuator for multimodal adaptive personal heat management
Source: Sci Adv. 2021 Dec 15;7(51):eabj7906. doi: 10.1126/sciadv.abj7906 (PMC8673776; doi:10.1126/sciadv.abj7906)
Supplement: Supplementary file 1 — Supplementary Text Figs. S1 to S13 Tables S1 and S2 References [file sciadv.abj7906_sm.pdf]

Science Advances
AAAS

Supplementary Materials for

Metalized polyamide heterostructure as a moisture-responsive actuator for multimodal adaptive personal heat management

Xiuqiang Li, Boran Ma, Jingyuan Dai, Chenxi Sui, Divya Pande, David R. Smith, L. Catherine Brinson*, Po-Chun Hsu*

*Corresponding author. Email: cate.brinson@duke.edu (L.C.B.); pochun.hsu@duke.edu (P.-C.H.)

Published 15 December 2021, *Sci. Adv.* **7**, eabj7906 (2021)
DOI: 10.1126/sciadv.abj7906

This PDF file includes:

Supplementary Text
Figs. S1 to S13
Tables S1 and S2
References

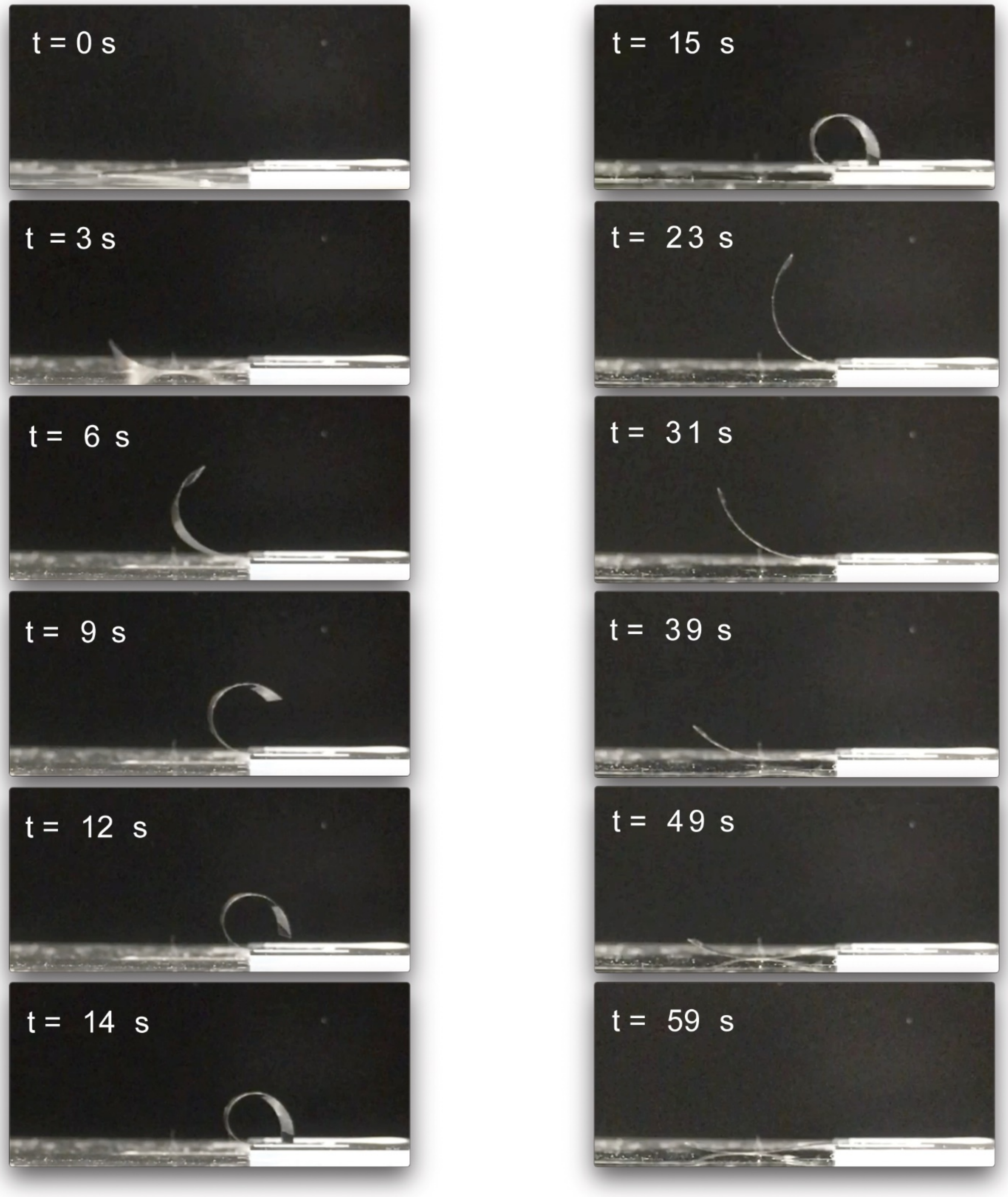

Fig. S1. Images of bending/recovering motion of nylon-Ag50 actuator over time. Photo credits: Jingyuan Dai, Duke University.

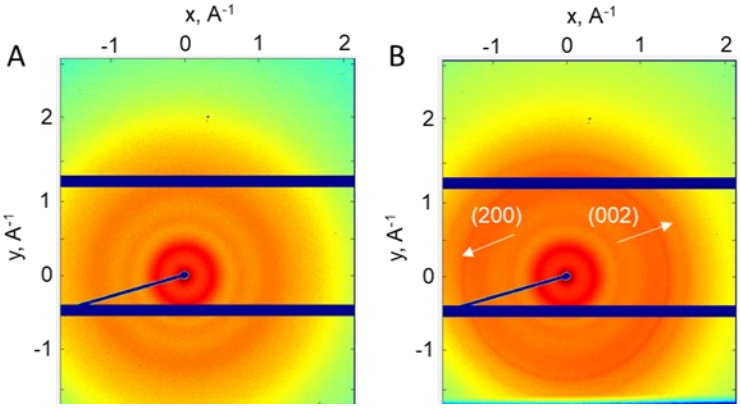

Fig. S2. **Two-dimensional WAXD analysis of nylon.** Two-dimensional WAXD patterns of the Kapton tape substrate (A) Kapton/nylon/Kapton (B).

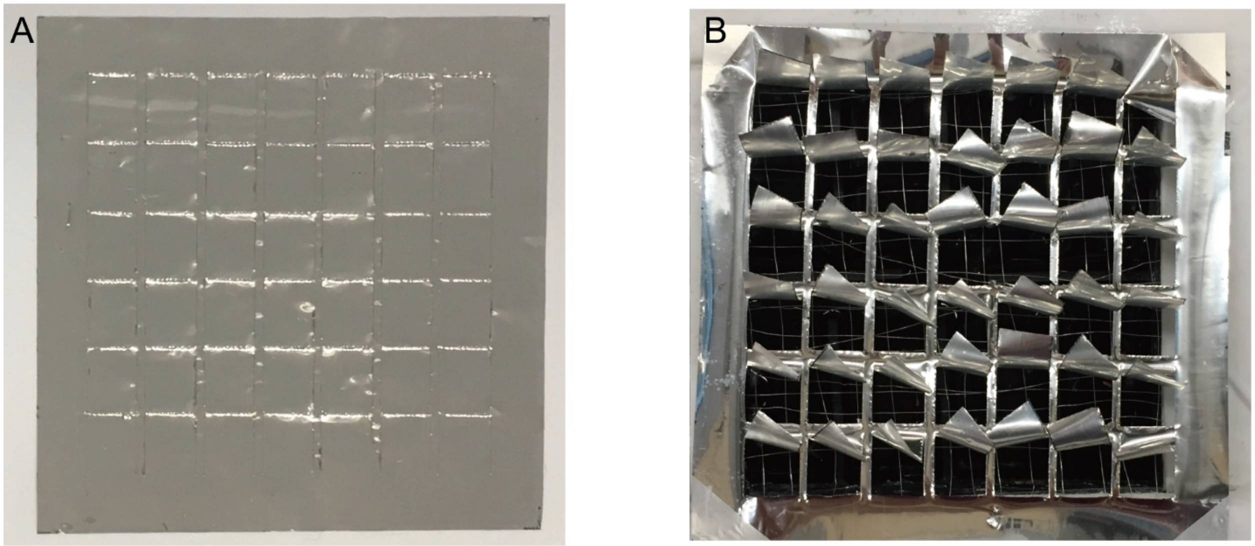

Fig. S3. The nylon/Ag film at different status. (A) nylon-Ag film with flaps closed, (B) Nylon Ag with flaps open. Photo credits: (A and B) Xiuqiang Li, Duke University.

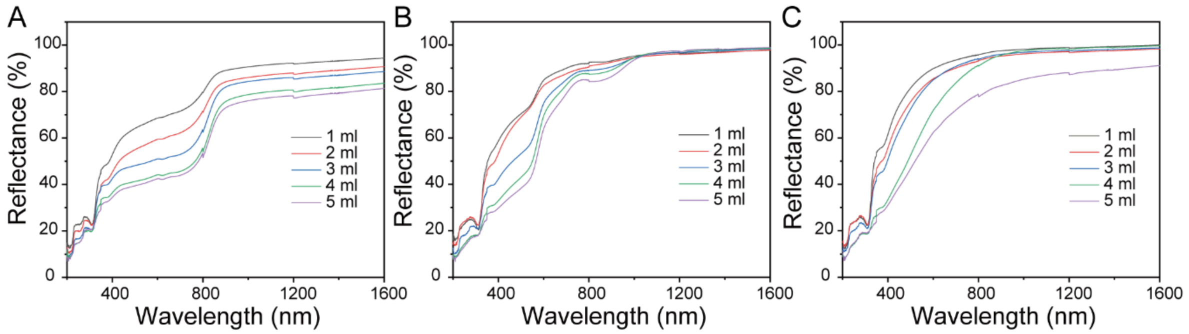

Fig. S4. The visible properties of wearables. Visible reflectance of nylon/Ag wearable with different concentration of CuO nanoparticles/SEBS (A), Fe_2O_3 nanoparticles/SEBS (B) and Si nanoparticles/SEBS (C), respectively.

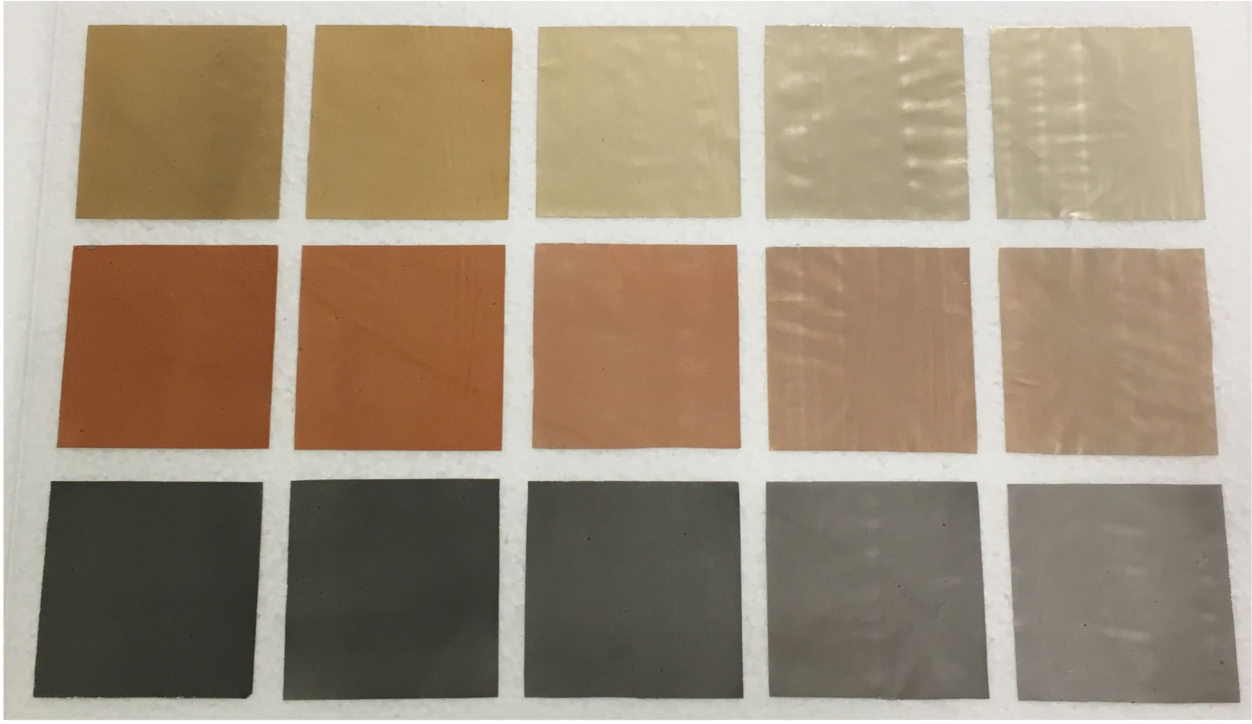

Fig. S5. Optical images of nylon-Ag wearable with different concentration. From left to right are 5 ml, 4 ml, 3 ml, 2 ml and 1 ml, respectively, of Si nanoparticles/SEBS (top), Fe_2O_3 nanoparticles/SEBS (middle) and CuO nanoparticles/SEBS (bottom), respectively. Photo credits: Jingyuan Dai, Duke University.

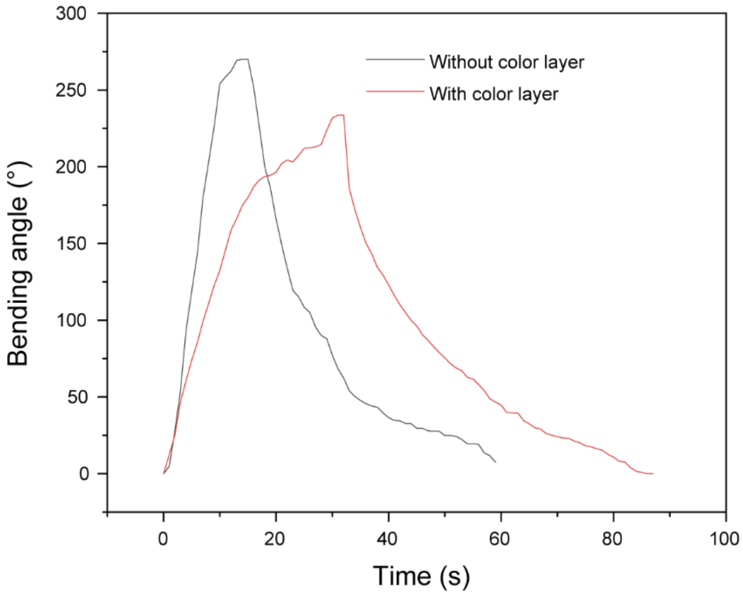

Fig. S6. Comparison of bending performance of nylon-Ag actuator with color layer (Fe_2O_3 nanoparticles/SEBS (5 ml)) and without color layer.

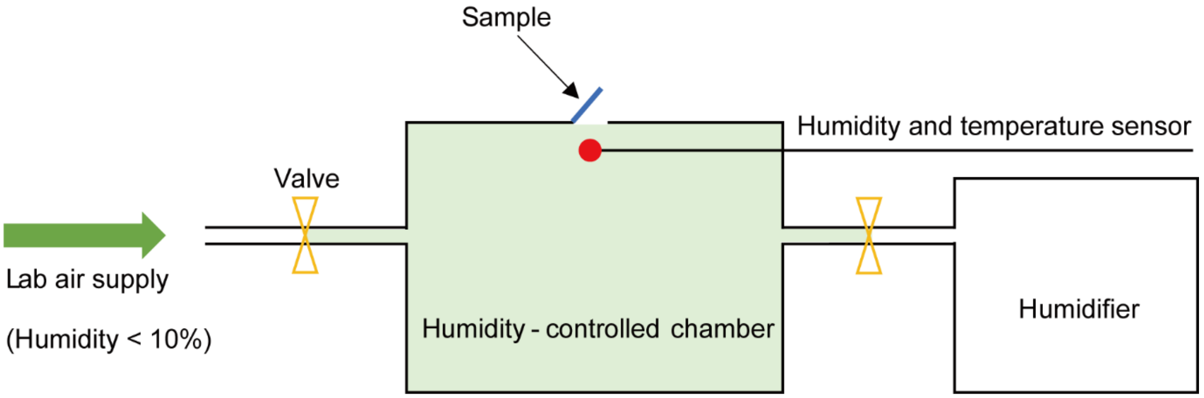

Fig. S7. Schematic of sample hygroscopic bending curvature measurement equipment.

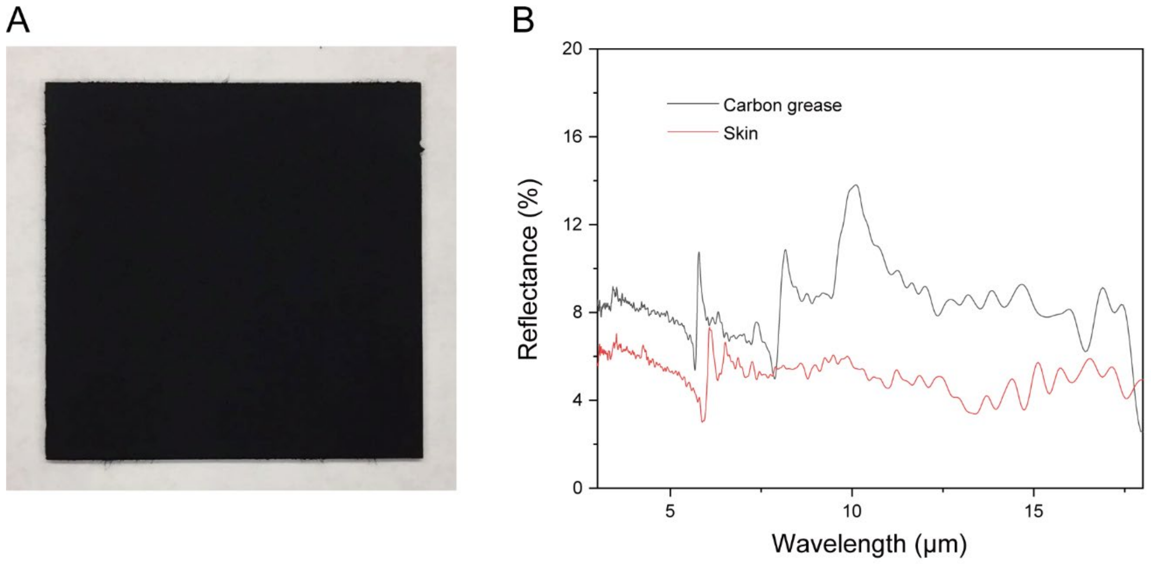

Fig. S8. The properties of skin and carbon grease. (A) Optical image of copper plate with carbon grease. (B) Mid-IR reflectance of skin and carbon grease. Photo credits: (A) Xiuqiang Li, Duke University.

Supplementary Table 1. Summary and comparison of passive moisture-responsive clothes reported in literature.

	**Materials**	**Tuning method**	**Flexibility**	**Open area for tuning**	**Radiation: low emissivity for heating**	**Radiation: high emissivity for cooling**	**Color design**
Zhang et al. (23)	Bimorph fibers with carbon nanotubes	Yarn	Folded	~ 35%	×	√	×
Fu et al. (25)	Hydrophobic polyethylene terephthalate and hydrophilic cellulose fibers	Yarn	Folded	~ 37%	×	√	×
Wang et al. (22)	Biohybrid film	Flap	-	Not given	×	√	×
Mu et al. (24)	Nafion	Flap	Flexible	Not given	×	√	√
Zhong et al. (21)	Nafion	Flap	Flexible	Not given	×	√	×
**Our work**	**Metallized nylon**	**Flap**	**Folded**	**~ 80%**	√	√	√

Note: the conduction and convection tuning and sweat evaporation tuning is largely dependent on porosity changes (4, 5).

Note 1. Mechanism analysis of the deposited silver nanolayer on the hygroscopic behavior of the nylon film.

In order to understand the effect of the deposited silver nanolayer on the hygroscopic behavior of the nylon film, mechanical analysis was carried out. As illustrated in Fig. S9, the schematic of the experimental setup, L_0 denotes the initial length of the flat nylon (bottom)/silver (top) bilayer. After exposed to moisture, the nylon layer expands, and the new stress-free length for the bottom layer becomes

$$L_0' = L_0 + \lambda L_0$$

(1)

where λ is the expansion ratio and $\lambda = \sqrt[3]{c_m \beta + 1} - 1$, with c_m being the concentration of moisture corresponding to the humidity in experiments and β as the expansion coefficient of nylon after water absorption. In this manner, different relative humidity results in different moisture concentration in the film, leading to changes in expansion and therefore bending. At position x along the thickness direction ($x = 0$ at the top surface of silver layer), the axial normal stresses σ_1 and σ_2 are given by

$$\sigma_1 = E_1 \left(\frac{\alpha(R + x)}{L_0} - 1 \right),$$
$$\sigma_2 = E_2 \left(\frac{\alpha(R + x)}{L_0 + \lambda L_0} - 1 \right).$$

(2)

where E_1 and E_2 are modulus of silver and nylon layers, respectively, α and R are the arc angle and the radius of curvature of the top surface of silver layer at equilibrium, respectively. α and R are unknowns we are solving. When the bilayer is at equilibrium, both the net forces and moments are zero, which gives us

$$\int_0^{t_1} \sigma_1 dx + \int_{t_1}^{t_1+t_2} \sigma_2 dx = 0,$$
$$\int_0^{t_1} \sigma_1 x dx + \int_{t_1}^{t_1+t_2} \sigma_2 x dx = 0,$$

(3)

where t_1 and t_2 are thickness of silver and nylon layers, respectively (34). Substituting Eq 2 into Eq 3 and solving the set of equations in Eq 3 give the absolute value of the radius of curvature at equilibrium:

$$|R| = \frac{A(1 - \lambda)t_1^4 + 2(2 + \lambda)t_1^3 t_2 + 3(2 + \lambda)t_1^2 t_2^2 + 4t_1 t_2^3 + \frac{1}{A} t_2^4}{6\lambda t_1 t_2 (t_1 + t_2)}$$

(4)

where $A = E_1/E_2$. A smaller value of $|R|$ corresponds to bending to a larger extent. The above equation provides a numerical explanation of the impact that depositing a thin layer of material with much higher modulus than the nylon film has on the bending behavior of the nylon film. For example, with a thin nanolayer of silver (Young's modulus ~83 GPa (32)) added, A~138.3 with the nylon film's Young's modulus being ~0.6 GPa as measured in experiments with water absorbed. Therefore, the contribution from the t_2 term is decreased by ~140 times, while the

contribution from the t_1 term is multiplied by ~140 times; since $t_2 \gg t_1$, the overall effect is a reduction in the radius of curvature, *i.e.*, an augmented bending of the nylon film. From a physical sense, the top layer of silver coating undergoes expansion at the interface in contact with the nylon film, whereas the top surface of the nylon film undergoes contraction. Therefore, a high Young's modulus of the coating layer gives rise to a smaller amount of strain at the interface resulting in an increase in bending.

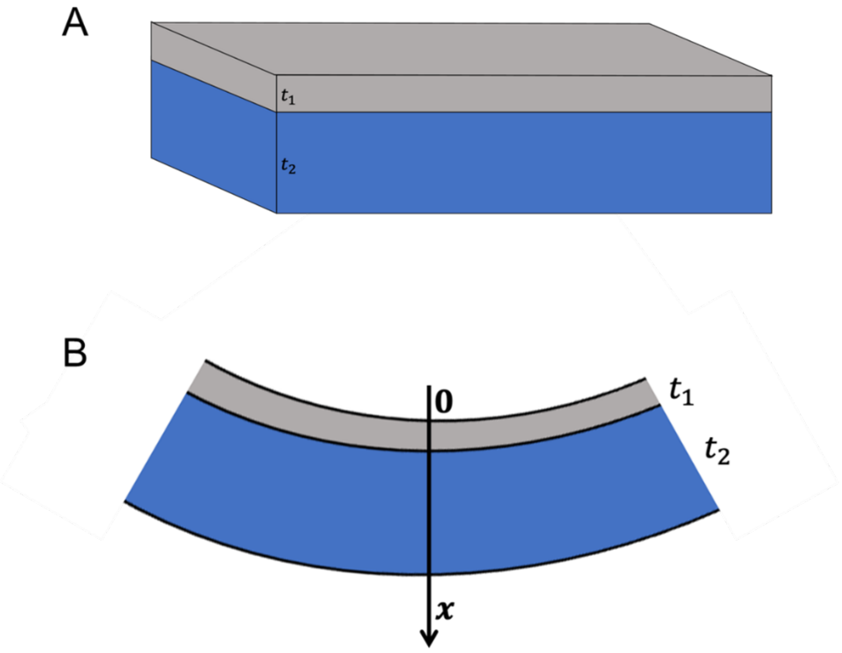

Fig. S9. Schematics of Nylon-Ag bilayer, t_1 and t_2 denote the thickness of silver and nylon, respectively. (A). the bilayer film before moisture source is turned on; (B). the bent bilayer film at equilibrium.

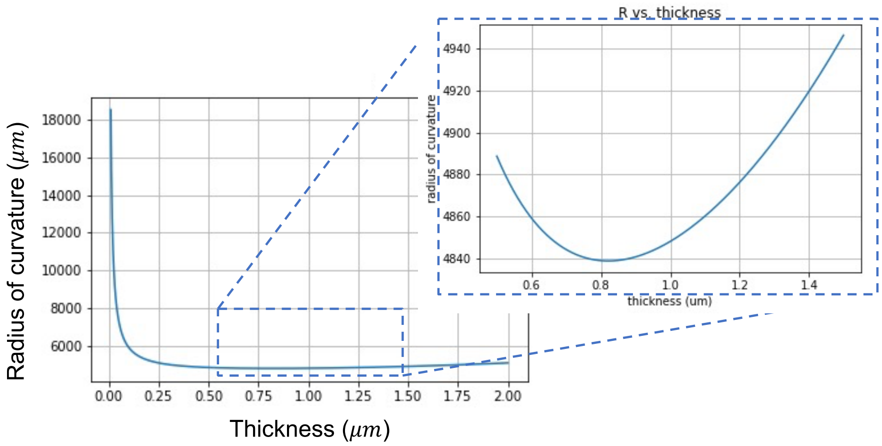

Fig. S10. **Radius of curvature of nylon-Ag bilayer film shows dependence on the thickness of the silver layer.** Zoomed-in subplot shows a non-monotonic trend.

The analytical result from Eq. 4 shows a non-monotonic trend in the thickness-dependent hygroscopic behavior of the nylon film (see Fig. S10). So far uniform water concentration, therefore, uniform strain is assumed in the model, which is unlikely due to experimental observations. Next, we incorporate gradient profile along the thickness direction of the film into the model. And the stress-free length for the bottom layer becomes

$$L_0' = L_0 + \lambda(x)L_0$$

(5)

where the expansion ratio λ is a function of x. A variety of gradient profiles (as shown in Fig. S11) obtained from solving diffusion equations were plugged in and calculated the relationship between radius of curvature of the bilayer film bending and thickness of the silver layer.

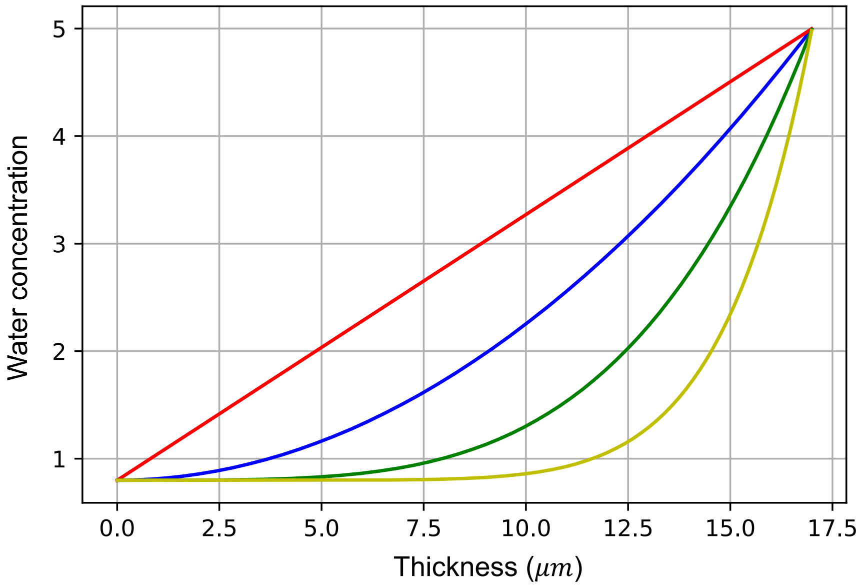

Fig. S11. **Concentration gradient profile along the thickness direction of the nylon film due to water absorption and diffusion.** Analytical forms of the curves are: red curve: $c = ax + b$, blue curve: $c = ax^2 + b$, green curve: $c = ax^4 + b$, yellow curve: $c = ax^8 + b$. These analytical forms are fitting equations for solutions of diffusion equations.

Substituting Eq. 5 with the gradient profiles shown in Fig. S11 into Eq. 2 and Eq. 3 yields the radius of curvature against the thickness of the silver layer. As shown in Fig. S12, the non-monotonic trend of the thickness dependence of radius of curvature of the bilayer film remains. Another trend is observed in Fig. S12, as the water concentration gradient profile changes from linear profile (red curve in Fig. S11) to a gradient profile described by the power law with a power of 8 (yellow curve in Fig. S11), the optimum thickness of the silver layer shifts to a smaller value (~530 nm) then shifts back.

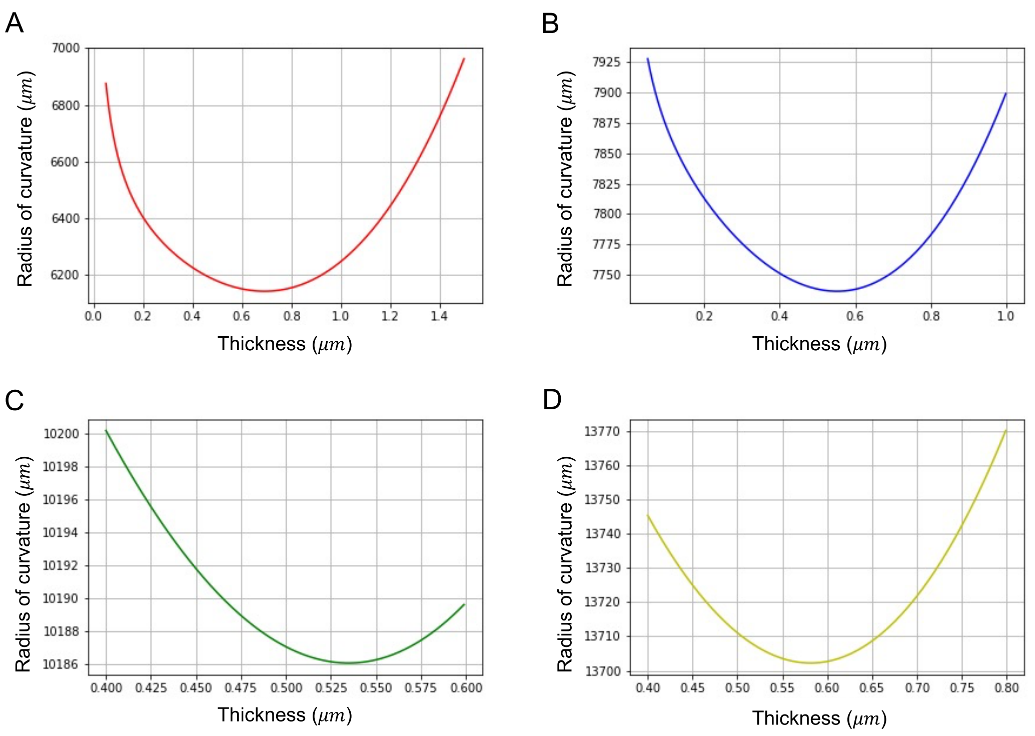

Fig. S12. **Radius of curvature of nylon-Ag bilayer film with various water concentration gradient profiles in the nylon layer against the thickness of the Ag layer.** The subplots correspond to the gradient profiles in Fig. S11.

The analytical model described above, when gradient concentration of water within in the nylon film is applied, yields the optimum thickness of the silver layer that is closer to the experimental result observed. However, it still shows discrepancy from the experimental case, where the bilayer structure with a 50 nm silver coating is shown to have the most pronounced bending angle (lowest radius of curvature). While it is possible that the Young's modulus of the electron beam evaporation deposited Ag film in these experiments differs from the thin Ag films of reference 32 also created using vapor deposition, any discrepancy would be small and insufficient to impact the analytical findings. More important to the discrepancy are fundamental limitations of the simplified analytical model in transmitting shear forces. While the analytical model has been validated by finite element analysis (FEA) simulations of a two-dimensional bilayer structure for the cases where the two layers have similar Young's moduli (e.g. 83 GPa and 20 GPa), for cases where the two layers have a larger Young's modulus mismatch, the analytical model does not fully capture the silver layer thickness dependence of the bending behavior of the bilayer structure. The lack of accuracy of the analytical model arises from omitting the shear force in between the two layers, which becomes more prevalent as the mismatch of the moduli of the two layers increases. Therefore, FEA was performed to further investigate the different bending behaviors of the film under different relative humidity values.

A series of FEA simulations of two-dimensional bilayer structures that characterize the mechanical properties of both nylon and silver layers were carried out using ABAQUS. The thermal expansion analysis included in ABAQUS was used as an analogy for the hygroscopic behavior of the nylon

film. One end of the bilayer film is fixed, just as the experimental set-up. Another boundary condition is a constant temperature (in analogy to relative humidity in experiments) applied to the bottom surface of the bilayer structure, the temperature field across the thickness direction of the nylon film follows the gradient profile as the green line shown in Fig. S11. Table. S2 summarizes the displacements of the tip of the free end of the nylon-Ag bilayer film from FEA simulations (Fig. S13). As the Ag layer thickness decreases from 50 nm to 40 nm, the bilayer film starts showing slight reduction in bending. To avoid dramatically increasing computation times as Ag layer thickness decreases further, Nylon-Ag20 was not simulated. In addition, Young's modulus of Ag film is expected to decrease when the thickness is reduced below 50 nm (1), this also leads to a smaller bending angle for Nylon-Ag20 based on the analytical modeling (Eq. 4). Therefore, Nylon-Ag50 leads to the largest bending angle, aligning with the optimum thickness observed in experiments.

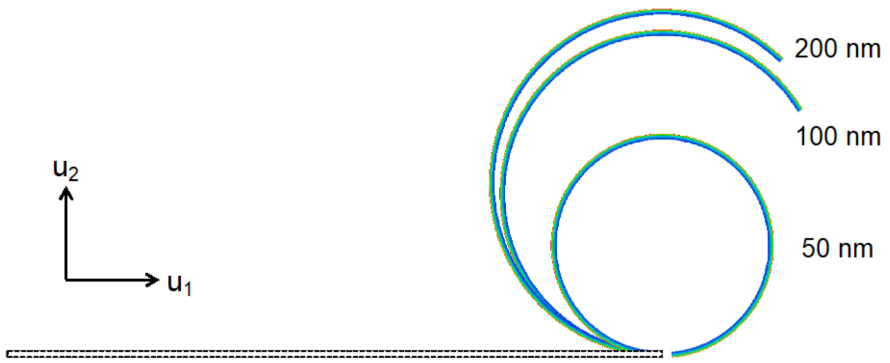

Fig. S13. FEA simulations of nylon-Ag bilayer film with various Ag thicknesses.

Supplementary Table 2. Summary of the displacements of tip of the free end of nylon-Ag film.

	u_1 (mm)	u_2 (mm)
Nylon-Ag40	2.03	0
Nylon-Ag50	2.03	0
Nylon-Ag100	2.41	0.73
Nylon-Ag200	2.36	0.88

REFERENCES AND NOTES

1. Department of Energy, Heating and Cooling; www.energy.gov/heating-cooling.

2. D. Ürge-Vorsatz, O. Lucon, H. Akbari, P. Bertoldi, L. F. Cabeza, N. Eyre, A. Gadgil, D. Harvey, Y. Jiang, E. Liphoto, S. Mirasgedis, S. Murakami, J. Parikh, C. Pyke, M. Vilariño, Chapter 9: Buildings, in *Climate Change 2014: Mitigation* (Intergovernmental Panel on Climate Change, 2014).

3. X. Li, W. Xie, C. Sui, P.-C. Hsu, Multispectral thermal management designs for net-zero energy buildings. *ACS Mater. Lett.* **2**, 1624–1643 (**2020**).

4. L. Peng, B. Su, A. Yu, X. Jiang, Review of clothing for thermal management with advanced materials. *Cellulose* **26**, 6415–6448 (2019).

5. Y. Peng, Y. Cui, Advanced textiles for personal thermal management and energy. *Joule* **4**, 724–742 (2020).

6. A. Ghahramani, K. Zhang, K. Dutta, Z. Yang, B. Becerik-Gerber, Energy savings from temperature setpoints and deadband: Quantifying the influence of building and system properties on savings. *Appl. Energy* **165**, 930–942 (2016).

7. Y. Cui, H. Gong, Y. Wang, D. Li, H. Bai, A thermally insulating textile inspired by polar bear hair. *Adv. Mater.* **30**, e1706807 (2018).

8. Z. Liu, J. Lyu, D. Fang, X. Zhang, Nanofibrous Kevlar aerogel threads for thermal insulation in harsh environments. *ACS Nano* **13**, 5703–5711 (2019).

9. T. Gao, Z. Yang, C. Chen, Y. Li, K. Fu, J. Dai, E. M. Hitz, H. Xie, B. Liu, J. Song, B. Yang, L. Hu, Three-dimensional printed thermal regulation textiles. *ACS Nano* **11**, 11513–11520 (2017).

10. P.-C. Hsu, X. Li, Photon-engineered radiative cooling textiles. *Science* **370**, 784–785 (2020).

11. J. K. Tong, X. Huang, S. V. Boriskina, J. Loomis, Y. Xu, G. Chen, Infrared-transparent visible-opaque fabrics for wearable personal thermal management. *ACS Photonics* **2**, 769–778 (2015).

12. P.-C. Hsu, A. Y. Song, P. B. Catrysse, C. Liu, Y. Peng, J. Xie, S. Fan, Y. Cui, Radiative human body cooling by nanoporous polyethylene textile. *Science* **353**, 1019–1023 (2016).

13. Y. Peng, J. Chen, A. Y. Song, P. B. Catrysse, P.-C. Hsu, L. Cai, B. Liu, Y. Zhu, G. Zhou, D. S. Wu, H. R. Lee, S. Fan, Y. Cui, Nanoporous polyethylene microfibres for large-scale radiative cooling fabric. *Nat. Sustain.* **1**, 105–112 (2018).

14. P.-C. Hsu, C. Liu, A. Y. Song, Z. Zhang, Y. Peng, J. Xie, K. Liu, C.-L. Wu, P. B. Catrysse, L. Cai, S. Zhai, A. Majumdar, S. Fan, Y. Cui, A dual-mode textile for human body radiative heating and cooling. *Sci. Adv.* **3**, e1700895 (2017).

15. X. Yue, T. Zhang, D. Yang, F. Qiu, G. Wei, H. Zhou, Multifunctional Janus fibrous hybrid membranes with sandwich structure for on-demand personal thermal management. *Nano Energy* **63**, 103808 (2019).

16. L. Lao, D. Shou, Y. S. Wu, J. T. Fan, "Skin-like" fabric for personal moisture management. *Sci. Adv.* **6**, eaaz0013 (2020).

17. X. Wang, Z. Huang, D. Miao, J. Zhao, J. Yu, B. Ding, Biomimetic fibrous Murray membranes with ultrafast water transport and evaporation for smart moisture-wicking fabrics. *ACS Nano* **13**, 1060–1070 (2019).

18. Y. Wang, X. Liang, H. Zhu, J. H. Xin, Q. Zhang, S. Zhu, Reversible water transportation diode: Temperature-adaptive smart Janus textile for moisture/thermal management. *Adv. Funct. Mater.* **30**, 1907851 (2019).

19. X. Yu, Y. Li, X. Wang, Y. Si, J. Yu, B. Ding, Thermoconductive, moisture-permeable, and superhydrophobic nanofibrous membranes with interpenetrated boron nitride network for personal cooling fabrics. *ACS Appl. Mater. Interfaces* **12**, 32078–32089 (2020).

20. B. Dai, K. Li, L. Shi, X. Wan, X. Liu, F. Zhang, L. Jiang, S. Wang, Bioinspired Janus textile with conical micropores for human body moisture and thermal management. *Adv. Mater.* **31**, e1904113 (2019).

21. Y. Zhong, F. Zhang, M. Wang, C. J. Gardner, G. Kim, Y. Liu, J. Leng, S. Jin, R. Chen, Reversible humidity sensitive clothing for personal thermoregulation. *Sci. Rep.* **7**, 44208 (2017).

22. W. Wang, L. Yao, C. Y. Cheng, T. Zhang, H. Atsumi, L. Wang, G. Wang, O. Anilionyte, H. Steiner, J. Ou, K. Zhou, C. Wawrousek, K. Petrecca, A. M. Belcher, R. Karnik, X. Zhao, D. I. C. Wang, H. Ishii, Harnessing the hygroscopic and biofluorescent behaviors of genetically tractable microbial cells to design biohybrid wearables. *Sci. Adv.* **3**, e1601984 (2017).

23. X. A. Zhang, S. Yu, B. Xu, M. Li, Z. Peng, Y. Wang, S. Deng, X. Wu, Z. Wu, M. Ouyang, Y. H. Wang, Dynamic gating of infrared radiation in a textile. *Science* **363**, 619–623 (2019).

24. J. Mu, G. Wang, H. Yan, H. Li, X. Wang, E. Gao, C. Hou, A. T. C. Pham, L. Wu, Q. Zhang, Y. Li, Z. Xu, Y. Guo, E. Reichmanis, H. Wang, M. Zhu, Molecular-channel driven actuator with considerations for multiple configurations and color switching. *Nat. Commun.* **9**, 590 (2018).

25. K. Fu, Z. Yang, Y. Pei, Y. Wang, B. Xu, Y. H. Wang, B. Yang, L. Hu, Designing textile architectures for high energy-efficiency human body sweat- and cooling-management. *Adv. Fiber Mater.* **1**, 61–70 (2019).

26. N. S. Murthy, M. Stamm, J. P. Sibilia, S. Krimm, Structural changes accompanying hydration in nylon 6. *Macromolecules* **22**, 1261–1267 (1989).

27. K. Inoue, S. Hoshino, Swelling of nylon 6 film due to water sorption. *J. Polym. Sci. Polym. Phys. Ed.* **14**, 1513–1526 (1976).

28. Y. Ge, R. Cao, S. Ye, Z. Chen, Z. Zhu, Y. Tu, D. Ge, X. Yang, A bio-inspired homogeneous graphene oxide actuator driven by moisture gradients. *Chem. Commun. (Camb.)* **54**, 3126–3129 (2018).

29. J. He, P. Xiao, J. Zhang, Z. Liu, W. Wang, L. Qu, Q. Ouyang, X. Wang, Y. Chen, T. Chen, Highly efficient actuator of graphene/polydopamine uniform composite thin film driven by moisture gradients. *Adv. Mater. Interfaces* **3**, 1600169 (2016).

30. J. Cao, Z. Zhou, Q. Song, K. Chen, G. Su, T. Zhou, Z. Zheng, C. Lu, X. Zhang, Ultrarobust $Ti_3C_2T_x$ MXene-based soft actuators *via* bamboo-inspired mesoscale assembly of hybrid nanostructures. *ACS Nano* **14**, 7055–7065 (2020).

31. IR Spectrum Table & Chart; https://www.sigmaaldrich.com/technical-documents/articles/biology/ir-spectrum-table.html.

32. H. Mizubayashi, J. Matsuno, H. Tanimoto, Young's modulus of silver films. *Scr. Mater.* **41**, 443–448 (1999).

33. L. Cai, Y. Peng, J. Xu, C. Zhou, C. Zhou, P. Wu, D. Lin, S. Fan, Y. Cui, Temperature regulation in colored infrared-transparent polyethylene textiles. *Joule* **3**, 1478–1486 (2019).

34. C. Li, Y. Xue, M. Han, L. C. Palmer, J. A. Rogers, Y. Huang, S. I. Stupp, Synergistic photoactuation of bilayered spiropyran hydrogels for predictable origami-like shape change. *Matter* **4**, 1377–1390 (2021).
